# Allelic variation of a clubroot resistance gene (*Crr1a*) in Japanese cultivars of Chinese cabbage (*Brassica rapa* L.)

**DOI:** 10.1270/jsbbs.21040

**Published:** 2022-03-05

**Authors:** Katsunori Hatakeyama, Shota Yuzawa, Kaoru Tonosaki, Yoshihito Takahata, Satoru Matsumoto

**Affiliations:** 1 Faculty of Agriculture, Iwate University, 3-18-8 Ueda, Morioka, Iwate 020-8550, Japan; 2 Institute of Vegetable and Floriculture Science, NARO, 3-1-1 Kannondai, Tsukuba, Ibaraki 305-8519, Japan; 3 Iwate Biotechnology Research Center, 22-174-4 Narita, Kitakami, Iwate 024-0003, Japan

**Keywords:** *Brassica rapa*, *Plasmodiophora brassicae*, clubroot resistance, nucleotide-binding domain leucine-rich repeat (NLR), allelic variation

## Abstract

Clubroot resistance (CR) is an important trait in Chinese cabbage breeding worldwide. Although *Crr1a*, the gene responsible for clubroot-resistance, has been cloned and shown to encode the NLR protein, its allelic variation and molecular function remain unknown. Here, we investigated the sequence variation and function of three *Crr1a* alleles cloned from six CR F_1_ cultivars of Chinese cabbage. Gain-of-function analysis revealed that *Crr1a^Kinami90_a^* isolated from the cv. ‘Kinami 90’ conferred clubroot resistance as observed for *Crr1a^G004^*. Because two susceptible alleles commonly lacked 172 amino acids in the C-terminal region, we investigated clubroot resistance in transgenic Arabidopsis harboring the chimeric *Crr1a*, in which 172 amino acids of the functional alleles were fused to the susceptible alleles. The fusion of the C-terminal region to the susceptible alleles restored resistance, indicating that their susceptibility was caused by the lack of the C-terminus. We developed DNA markers to detect the two functional *Crr1a* alleles, and demonstrated that the functional *Crr1a* alleles were frequently found in European fodder turnips, whereas they were rarely introduced into Japanese CR cultivars of Chinese cabbage. These results would contribute to CR breeding via marker-assisted selection and help our understanding of the molecular mechanisms underlying clubroot resistance.

## Introduction

Clubroot disease, caused by the soil-borne protozoan *Plasmodiophora brassicae* Wor., is a major concern in the production of cruciferous crops worldwide (Dixon 2009). Yield loss due to clubroot disease has been estimated to be approximately 50% in canola in Canada (Pageau *et al.* 2006) and 20%–30% in cruciferous crops in China (Chai *et al.* 2014). In Japan, this disease was first recorded in 1892 (Ikegami *et al.* 1981) and has become a major threat to *Brassica* vegetable production. Clubroot disease was observed in approximately 650 ha of Chinese cabbage fields in 2018. Because of the long-term survival potential of resting spores, it is difficult to control infection using agricultural practices such as crop rotation and liming. Therefore, breeding of clubroot-resistant (CR) cultivars is believed to be one of the most economical and environmental friendly methods for preventing clubroot infection (Diederichsen *et al.* 2009). Most of the F_1_ cultivars of Chinese cabbage released in Japan possess varying levels of clubroot resistance. However, variations in pathogenicity and virulence among the field populations of *P. brassicae* cause a breakdown of resistance on a part of the CR cultivars (Tanaka *et al.* 2006). Several differential tester sets have been reported by the studies of the pathotypes of single-spore isolates and field isolates (Buczacki *et al.* 1975, Hatakeyama *et al.* 2004, Some *et al.* 1996, Williams 1966). Four pathotypes (groups 1, 2, 3, and 4), from two CR F_1_ cultivars of Chinese cabbage as differential hosts, were identified using the classification system (Hatakeyama *et al.* 2004). This system has been used to assess the clubroot-infested fields in Japan.

Most of the sources of clubroot resistance were derived from European fodder turnips, and the CR genes derived from them have been introduced into Chinese cabbage (*B. rapa*), canola (*B. napus*), and *B. oleracea* crops by traditional breeding (Diederichsen *et al.* 2009). Genetic mapping of these resistance sources in *B. rapa* revealed the identification of more than 20 CR loci, some of which have been introduced into commercial F_1_ cultivars of Chinese cabbage (Chen *et al.* 2013, Chu *et al.* 2014, Hirai *et al.* 2004, Hirani *et al.* 2018, Karim *et al.* 2020, Kato *et al.* 2012, 2013, Laila *et al.* 2019, Matsumoto *et al.* 1998, 2012, Pang *et al.* 2018, Piao *et al.* 2004, Saito *et al.* 2006, Sakamoto *et al.* 2008, Suwabe *et al.* 2003, 2006, Yu *et al.* 2017). Because the clubroot resistance test for selection is laborious and time-consuming, marker-assisted selection is believed to be a powerful tool for resistance breeding. However, because of the differences among genetic resources and pathogens used for mapping, the precise location of most CR loci and their pathotype specificity remains unclear, which hinders effective breeding of CR cultivars of Chinese cabbage. Although numerous DNA markers linked to various CR loci have been reported, PCR analysis using these markers is insufficient for the detection of CR genes.

Among the CR genes identified in *B. rapa* chromosomes in recent times, the genes *Crr1a* and *CRa* have been cloned (Hatakeyama *et al.* 2013, 2017, Ueno *et al.* 2012). Cloning of CR genes leads to the development of gene-specific markers and is indispensable for increasing the efficiency and accuracy of marker-assisted selection of *B. rapa* crops. Identification of *CRb* (also referred to as *CRb^Kato^*) revealed that this gene is the same allele as the previously cloned *CRa* (Hatakeyama *et al.* 2017). PCR analysis using the SCAR marker, designed based on the sequence of the *CRa* gene, revealed that this gene is widely distributed in the CR cultivars of Chinese cabbage (Aruga *et al.* 2013). However, the allelic variation of CR genes in F_1_ cultivars of Chinese cabbage has not been well studied. Although the susceptible *Crr1a^A9709^* allele contained a retrotransposon-like insertion in exon 1, which was likely a cause of susceptibility, this insertion was not always a common feature in non-CR cultivars of Chinese cabbage (Hatakeyama *et al.* 2013). Moreover, information on the types and number of functional CR genes introduced in commercial cultivars of Chinese cabbage is still limited.

Cloned CR genes encode nucleotide-binding (NB) leucine-rich repeat (LRR) receptors (NLRs) carrying the Toll/interleukin-1 receptor (TIR) domain at the N terminus (Hatakeyama *et al.* 2013, 2017, Ueno *et al.* 2012). Plant NLRs induce disease resistance responses by recognizing effector proteins released by pathogens, either directly or indirectly, by monitoring the guradees or decoys (Adachi *et al.* 2019, Białas *et al.* 2018, Jones *et al.* 2016, Kourelis and van der Hoorn 2018). Some NLRs function as single genetic units for the recognition of pathogens and initiate immune signaling, whereas others work in pairs by sharing roles in recognition and signaling. The LRR domain of the NLR protein is involved in the direct or indirect recognition of the pathogen effectors (Adachi *et al.* 2019, Ma *et al.* 2020), whereas some NLRs that work in pairs contain an additional integrated domain (ID) required for effector recognition (Białas *et al.* 2018). However, functional analysis has not been performed, and the cloned CR genes and molecular mechanisms underlying clubroot resistance via CR genes remain unclear.

In this study, we investigated the function of three *Crr1a* alleles cloned from CR cultivars of Chinese cabbage and discovered another functional allele, which has the same pathotype specificity as *Crr1a^G004^*. Here, we showed that the C-terminal region is essential for the clubroot resistance conferred by Crr1a protein, through a domain-swapping experiment. Furthermore, we developed gene-specific markers for the functional *Crr1a* alleles and demonstrated the distribution of functional alleles in Japanese commercial F_1_ cultivars of Chinese cabbage and resistant European fodder turnips.

## Materials and Methods

### Plant materials and test for clubroot resistance

In this study, 48 CR and 12 non-CR commercial F_1_ cultivars of Chinese cabbage were used (see Supplemental Table 1). In addition, ‘Moonbeach’ (Musashino Seed Co., Ltd.) was used for cloning the *Crr1a* allele. The clubroot susceptible line A9709, resistant line G004, and resistant European fodder turnip varieties, ‘Siloga’, ‘Debera’, ‘Gelria R’, and ‘77b’ were used.

The *Plasmodiophora brassica* field isolate Ano-01 was used, and the pathotype belonged to group 4, as previously defined (Hatakeyama *et al.* 2004, 2013, 2017, Kato *et al.* 2012). The test for clubroot resistance in *Arabidopsis thaliana* and the evaluation of root symptoms were performed as described by Hatakeyama *et al.* (2013). Arabidopsis Col-0 was used as a susceptible control in each test to determine the virulence of the isolate.

### DNA extraction and cloning of genomic clones of *Crr1a* alleles

Genomic DNA was isolated using a DNeasy Plant Mini Kit (Qiagen, Germany). Amplification was carried out using Ex Taq HS polymerase (TaKaRa Bio, Japan) as follows: initial denaturation at 94°C was performed for 2 min followed by 30 cycles of amplification, including 10 s at 94°C, 30 s at 58°C, and 6 min at 72°C. The PCR product was ligated into the XL-TOPO vector (Thermo Fisher Scientific, USA) according to the manufacturer’s instructions. At least three clones were sequenced to verify the sequence.

### Vector construction and transformation

Genomic DNA fragments of the full-length *Crr1a* allele were amplified using KOD+ DNA polymerase (Toyobo, Japan), with the primers described in Supplemental Table 2, and inserted downstream of the *Crr1a^G004^* promoter sequence (Hatakeyama *et al.* 2013).

The chimeric *Crr1a* genes used in this study are shown in Supplemental Fig. 1. Genomic DNA fragments of protein-coding regions including TIR, NB, and LRR domains were amplified from *Crr1a^Hiroki_b^* and *Crr1a^Kiko85_a^*, and the C-terminal regions were amplified from the functional *Crr1a^G004^* and *Crr1a^Kinami90_a^* using the primer pairs listed in Supplemental Table 2. These two fragments were fused downstream of the *Crr1a^G004^* promoter of the cassette vector using the In-Fusion HD Cloning Kit (TaKaRa Bio). In addition, we constructed a chimeric gene in which the C-terminal region of *Crr1a^G004^* was replaced with that of *Crr1a^Kinami90_a^*. The sequence of the resultant gene constructs was confirmed by Sanger sequencing. Ligation of the gene cassette into the binary vector and transformation of *A. thaliana* were carried out as previously described (Hatakeyama *et al.* 2013).

### Detection of *Crr1a* alleles in F_1_ cultivars of Chinese cabbage and CR turnips

Genomic DNA was extracted using a DNeasy Plant 96 kit (Qiagen). Bulked DNA samples were generated from eight plants of each cultivar of Chinese cabbage. Genomic DNA isolated from 16 plants of each variety was used for the analysis of CR turnips. The primers used are shown in Supplemental Table 2. Amplification, using Ex Taq HS DNA polymerase (TaKaRa Bio), was performed by initial denaturation at 94°C for 2 min followed by 30 cycles of 10 s at 94°C, 30 s at 58°C, 30 s at 72°C, and a final extension for 3 min at 72°C. PCR fragments were separated on a 2% agarose gel.

## Results

### Sequence variation of *Crr1a* alleles

**Fig. 1.**
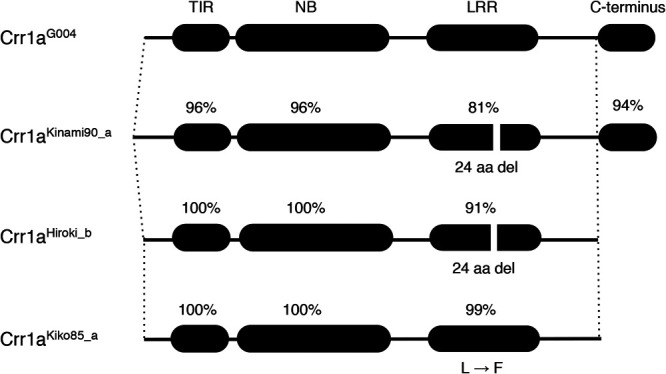
Schematic representation of the deduced protein sequences of *Crr1a* alleles. Amino acid sequence identities of TIR, NB, LRR domains, and C-terminal region against Crr1a^G004^ are indicated above each domain. The sequences of *Crr1a* alleles were deposited in the GenBank database (LC593254, LC593255, and LC593256).

Compared with *Crr1a^G004^*, the susceptible *Crr1a^A9709^* contained a 357-bp insertion in exon 1, leading to a lack of half of the TIR domain (Hatakeyama *et al.* 2013). PCR analysis using primers flanking this insertion (Crr1a_exon1_F and Crr1a_exon1_R) revealed the presence of a 347-bp PCR product in G004 and 704-bp in A9709. When this PCR analysis was applied to 48 CR cultivars of Chinese cabbage, the 347-bp PCR product for *Crr1a^G004^* was observed in 21 CR cultivars and 7 non-CR cultivars, whereas a 416-bp PCR product was detected in two CR cultivars (‘Kinami 90’ and ‘W-1117’). To analyze whether these differences are associated with the function of *Crr1a* alleles, we attempted to clone the full-length genomic DNA fragment from CR cultivars using PCR and obtained seven clones from six CR cultivars (SCR Hiorki, Kiko 86, Kinami 90, W-1116, W-1117, Moonbeach). Four types of alleles were identified based on sequence differences (Fig. 1).

Comparison of the predicted proteins of the four alleles showed that the TIR and NB domains were well conserved, while the LRR and C-terminal domains were variable (Supplemental Figs. 1–4). Of the two clones obtained from ‘Super CR Hiroki’, one was identical to *Crr1a^G004^* and another clone, which was also amplified for ‘Moonbech’, contained a 72-bp deletion and 898-bp insertion in exon 4 compared with *Crr1a^G004^*. The latter clone was named *Crr1a^Hiroki_b^*. The 72-bp deletion resulted in the lack of 24 amino acids in the LRR domain and the 898-bp insertion lead to the production of an immediate stop codon, resulting in the lack of 172 amino acids at the C-terminus (Supplemental Figs. 2–4). The *Crr1a^Kiko85_a^* allele derived from ‘Kiko 85’ and ‘W-1116’, had a 1-bp non-synonymous substitution, resulting in Leu to Phe substitution, and an 898-bp insertion in exon 4, as found in *Crr1a^Hiroki_b^*. The *Crr1a^Kinami90_a^*, which was derived from ‘Kinami 90’, contained lots of base substitutions and several insertion/deletions. The 416-bp PCR product amplified from ‘Kinami 90’ and ‘W-1117’ was caused by the insertion of 69-bp in exon 1. The predicted protein had 59 amino acid substitutions, two insertions of 6 amino acids and 17 amino acids in the N-terminal region upstream of the TIR domain, and 24 amino acid deletions in the LRR domain. Among the 59 amino acid substitutions, 1, 9, and 21 were found in the TIR, NB, and LRR domains, respectively.

### Functional analysis of *Crr1a* alleles using transgenic Arabidopsis

**Fig. 2.**
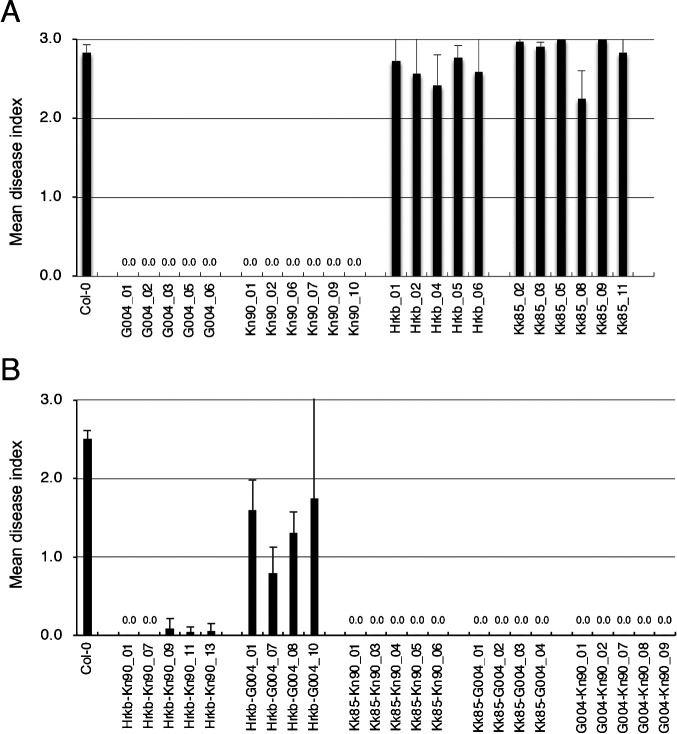
Resistance of the transgenic T_1_ lines carrying the genomic DNA fragments of *Crr1a* alleles (A) and the chimeric Crr1a genes (B) to Ano-01. A, G004, Kn90, Hrkb, and Kk85 indicate the T_1_ lines carrying *Crr1a^G004^*, *Crr1a^Kinami90_a^*, *Crr1a^Hiroki_b^*, and *Crr1a^Kiko85_a^*, respectively. B, Hrkb-Kn90, Hrkb-G004, Kk85-Kn90, Kk85-G004, and G004-Kn90 indicate the T_1_ lines carrying *Crr1a^Hrkb-Kn90^*, *Crr1a^Hrkb-G004^*, *Crr1a^Kk85-Kn90^*, *Crr1a^Kk85-G004^*, and *Crr1a^G004-Kn90^*, respectively (Supplemental Fig. 1). Mean disease indices (±SD) of the T_1_ lines were calculated based on the average of two to three clubroot resistance tests (~9 T_2_ plants per test).

**Fig. 3.**
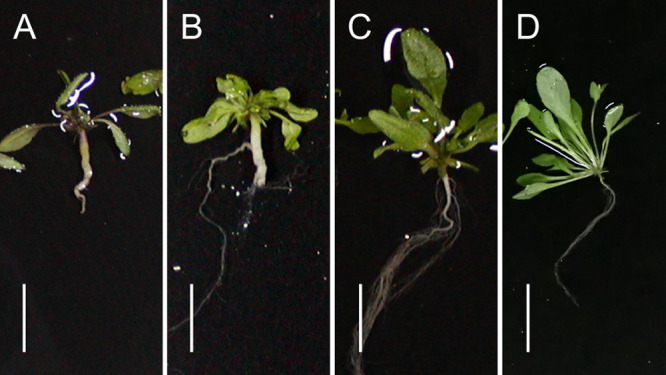
Typical root phenotype of the inoculated Arabidopsis wild type (A) and transgenic plants with *Crr1a^Kiko85_a^* (B), *Crr1a^Kinami90_a^* (C), and *Crr1a^Kiko85-G004^* (D). Scale bar indicates 10 mm.

To determine whether the three alleles cloned in this study were functional or not, genomic DNA fragments of *Crr1a^G004^*, *Crr1a^Hiroki_b^*, *Crr1a^Kiko85_a^*, and *Crr1a^Kinami90_a^* were transferred into the clubroot-susceptible Arabidopsis, Col-0, and T_2_ plants derived from independent T_1_ lines, which were then inoculated with the isolate Ano-01. Transgenic plants with *Crr1a^G004^* showed complete resistance against Ano-01, whereas those harboring *Crr1a^Hiroki_b^* and *Crr1a^Kiko85_a^* were fully susceptible (mean disease index >2.0, Figs. 2A, 3). Transgenic plants with *Crr1a^Kinami90_a^* showed high resistance (mean disease index = 0.0). Furthermore, we confirmed that *Crr1a^Kinami90_a^* conferred resistance against pathotypes 2 and 4, and showed susceptibility to pathotype 3, as observed for *Crr1a^G004^* (data not shown). These results indicated that *Crr1a^Kinami90_a^* is another functional allele.

The common feature of the two susceptible *Crr1a* alleles, *Crr1a^Hiroki_b^* and *Crr1a^Kiko85_a^*, is the lack of 172 amino acids at the C-terminus. To study whether the C-terminal region of the *Crr1a* gene is essential for conferring clubroot resistance, we constructed chimeric genes, in which the susceptible alleles were fused with the C-terminal region of the functional allele, and transferred them into Col-0 plants. We then examined the resistance of the transgenic plants with the chimeric *Crr1a* allele. All the transgenic plants harboring the chimeric *Crr1a* gene showed resistance against Ano-01, while the transgenic plants harboring the chimeric *Crr1a^Hiroki_b^* gene fused with the C-terminal region of *Crr1a^G004^* showed partial resistance (mean DI: 0.8–1.7) (Figs. 2B, 3). Furthermore, we constructed a chimeric *Crr1a* gene in which the C-terminal region of *Crr1a^G004^* was replaced with that of *Crr1a^Kinami90_a^* and evaluated the resistance of the transgenic plants. The resultant chimeric gene (*Crr1a^G004-Kn90^*) conferred resistance against Ano-01 (Fig. 2B). These results strongly indicate that the C-terminal region of the *Crr1a* gene could compensate the resistant phenotype in susceptible alleles lacking the C-terminus and therefore, is essential for clubroot resistance.

### Detection of functional *Crr1a* alleles in CR Chinese cabbage and CR turnips

**Fig. 4.**
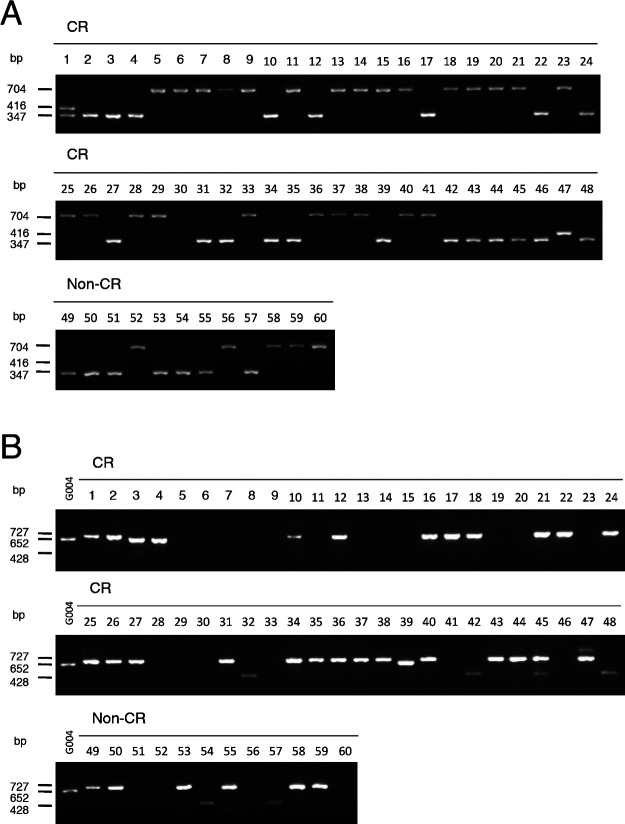
Detection of the functional alleles of *Crr1a* gene in 48 CR and 12 non-CR cultivars of Chinese cabbage. Details of the used cultivars used are indicated in Supplemental Table 1. A, Detection of the *Crr1a^Kinami90_a^* allele using the primers Crr1a_exon1_F and Crr1a_exon1_R. B, Detection of the *Crr1a^G004^* allele using the primers Crr1a_exon4_F, Crr1a_exon4_R1, and Crr1a_exon4_R2.

**Fig. 5.**
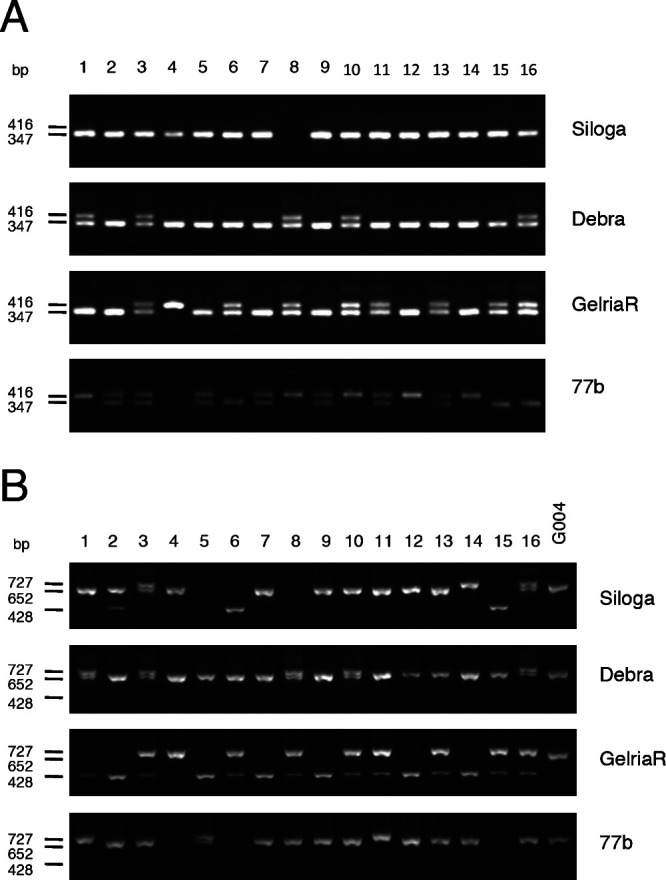
Detection of the functional alleles of *Crr1a* gene in 16 plants of European fodder turnips. A, Detection of the *Crr1a^Kinami90_a^* allele using the primers Crr1a_exon1_F and Crr1a_exon1_R. B, Detection of the *Crr1a^G004^* allele using the primers Crr1a_exon4_F, Crr1a_exon4_R1, and Crr1a_exon4_R2.

We investigated the presence of functional *Crr1a* alleles in commercial CR cultivars of Chinese cabbage. Because the positions of mutations were variable among the *Crr1a* alleles (Supplemental Fig. 6), we considered it reasonable to develop allele-specific markers for each functional allele. We designed primer pairs to detect a 69-bp insertion in exon 1 that is found only in the *Crr1a^Kinami90_a^* allele. PCR analysis using the primers Crr1a_exon1_F and Crr1a_exon1_R, could detect a 416-bp PCR product in ‘Kinami 90’ and 347-bp in G004 (Supplemental Table 2, Supplemental Fig. 6). Because there is a 357-bp insertion in exon 1 of the susceptible A9709, a 704-bp PCR product was detected in A9709. PCR analysis of 48 CR and 12 non-CR cultivars revealed that the 416-bp PCR product was detected only in ‘Kinami 90’ and ‘W-1117’, but not in any of the non-CR cultivars (Fig. 4A). Cloning of the full-length *Crr1a* gene from ‘W-1117’ confirmed that this cultivar possessed *Crr1a^Kinami90_a^*. These results indicated that the exon 1 marker (Crr1a_exon1_F and Crr1a_exon1_R) can be useful for specifically detecting functional *Crr1a^Kinami90_a^*. The 347-bp PCR product for *Crr1a^G004^* was observed in 21 CR cultivars and 7 non-CR cultivars, and the 704-bp PCR product was detected in 26 CR cultivars. It is likely that ‘Kinami 90’ and ‘W-1117’ possessed *Crr1a^Kinami90_a^* in heterozygous and homozygous forms, respectively. Furthermore, we tested this marker in the CR turnips. The 416-bp product, corresponding to *Crr1a^Kinami90_a^*, was detected in more than one plant from ‘Debra’, ‘Gelria R’, and ‘77b’, but in none from ‘Siloga’ (Fig. 5A).

To develop a marker to discriminate between *Crr1a^G004^* and *Crr1a^Hiroki_b^*, we focused on sequence differences in the 3ʹ region of *Crr1a* alleles and designed three primers: Crr1a_exon4_F, Crr1a_exon4_R1, and Crr1a_exon4_R2 (Supplemental Table 2, Supplemental Fig. 6). Crr1a_exon4_F and Crr1a_exon4_R2 flank the 898-bp insertion found in the susceptible *Crr1a^Hiroki_b^* and *Crr1a^Kiko85_a^* and Crr1a_exon4_R1 is located in the reverse orientation within this insertion. A 652-bp PCR product was expected to be obtained from G004. A 428-bp PCR product flanked by Crr1a_exon4_F and Crr1a_exon4_R1 was expected to be detected in *Crr1a^Hiroki_b^* and *Crr1a^Kiko85_a^*, and no PCR products were expected from the susceptible A9709 because this line has more than 5-kb insertion in exon 4 (Hatakeyama *et al.* 2013) (Supplemental Fig. 6). A 727-bp PCR product was detected in ‘Kinami 90’, which was attributed to a 75-bp insertion located immediately downstream of the stop codon of *Crr1a*. The PCR analysis of 48 CR and 12 non-CR cultivars of Chinese cabbage revealed that a 652-bp product was detected in three cultivars, ‘Akimeki’, ‘SCR Hiroki’, and ‘SCR Kimi 85’ (Fig. 4B). The 428-bp PCR product was detected in 5 CR cultivars, ‘SCR Hiroki’, ‘Chiyobuki 70’, ‘Kiko 85’, ‘Kiai 65’, and ‘W-1116’. PCR analysis of CR turnips revealed that the 652-bp product corresponding to *Crr1a^G004^* was detected in more than one plant from ‘Siloga’ and ‘77b’, and in all plants from ‘Debra’, whereas none was detected from ‘Gelria R’. Surprisingly, the 428-bp PCR product for *Crr1a^Hiroki_b^* was detected in most of the plants from ‘Gelria R’ (Fig. 5B).

## Discussion

In this study, we investigated the sequence variation and function of three *Crr1a* alleles cloned from six CR F_1_ cultivars of Chinese cabbage. The TIR and NB domains of the predicted proteins encoded by these alleles were well conserved, but the LRR domain and C-terminus were variable (Fig. 1). We demonstrated that *Crr1a^Kinami90_a^* conferred resistance to the clubroot isolate, while the other two did not (Fig. 2A). The two susceptible alleles, *Crr1a^Hiroki_b^* and *Crr1a^Kiko85_a^*, showed high sequence identity to the functional allele *Crr1a^G004^* though they commonly contained a 898-bp insertion in exon 4, resulting in a lack of 172 amino acids at the C-terminus (Fig. 1, Supplemental Fig. 6). Gain-of-function analysis using the chimeric *Crr1a* gene revealed that fusion of the C-terminal region derived from the functional alleles could restore the ability of clubroot resistance in the two susceptible alleles (Figs. 2B, 3). This result suggests that the lack of a C-terminal region is a cause of the loss of resistance.

The significance of the C-terminal domain has been reported for several NLR proteins. Truncation and mutation of the C-terminal non-LRR region of the P2 flax rust resistance protein causes loss of function (Dodds *et al.* 2001). This domain is shared by other TIR-NLR disease resistance proteins such as RPS4, RPP1, and RPP5 in Arabidopsis and the N in tobacco, suggesting a possible role in specificity determination. Some NLRs possess an integrated domain at the C-terminus, which plays a role in effector recognition and NLR activation. Rice RGA5 possesses an HMA domain for Avr recognition at the C-terminus (Cesari *et al.* 2013). The Arabidopsis RRS1-R protein recognizes effectors via the integrated WRKY domain at the C-terminus, and its C-terminal region is also involved in the regulation of active and inactive forms of this protein (Guo *et al.* 2020, Sarris *et al.* 2015). Although we could not find any known motifs and domains in this region, these findings suggest that the C-terminal region of the Crr1a protein has a similar function. Recent findings suggest that some NLRs form complexes as singletons, or in pairs to activate immunity (Adachi *et al.* 2019). Therefore, we cannot exclude the possibility that the lack of C-terminus disrupts the formation of such complexes, resulting in the loss of resistance.

The resistance response of the susceptible *Crr1a^Hiroki_b^* allele depended on the fused C-terminal region of the functional allele. The chimeric *Crr1a^Hrkb-Kn90^* conferred complete resistance, whereas *Crr1a^Hrkb-G004^*, in which *Crr1a^Hiroki_b^* was fused with the C-terminal region of *Crr1a^G004^*, resulted in partial resistance (Fig. 2B). The deduced chimera Crr1a^Hrkb-G004^ protein was almost identical to the functional Crr1a^G004^ except for the 24-aa deletion in the LRR domain, which might also be involved in inducing resistance. Arabidopsis TIR-NLR receptor RPP1 confers strain-specific immunity through the recognition of *Hyaloperonospora arabidopsidis* effector ATR1. RPP1 was reported to be activated by direct binding of the effector to the C-terminal JID domain and LRRs, leading to the induction of an RPP1 tetrameric assembly required for host cell death (Ma *et al.* 2020). Although Crr1a^Kinami90_a^, which also contained a 24-aa deletion in LRR, could confer complete resistance (Fig. 2A), this allele conferred complete resistance. It is possible that the difference in resistance between Crr1a^Kinami90_a^ and Crr1a^Hrkb-G004^ is due to the amino acid sequence differences found in the LRR and C-terminal regions of the Crr1a protein (Fig. 1, Supplemental Figs. 1, 5). In the case of Crr1a-mediated signaling, the combination of LRR with 24-aa deletion and C-terminus of Crr1a^G004^ in Crr1a^Hrkb-G004^ may affect the affinity of effector binding or cause a conformational change in the NLR immune receptor complex, resulting in incomplete resistance. Further investigation using the chimeric or mutagenized *Crr1a* gene would be necessary to understand the cause of partial resistance conferred by the chimeric *Crr1a^Hrkb-G004^* and provide insights into the function of Crr1a in clubroot resistance.

Because the clubroot test for selection is laborious and time-consuming, marker-assisted selection is considered the most effective way to develop cultivars with high levels of resistance (Diederichsen *et al.* 2009). In this study, we found another functional *Crr1a* allele, *Crr1a^Kinami90_a^*, in addition to *Crr1a^G004^*. Based on the sequence comparison, we successfully developed PCR-based markers to identify *Crr1a^Kinami90_a^* and *Crr1a^G004^* alleles. Genotyping analysis of the 48 CR F_1_ cultivars revealed that five of the cultivars possessed the functional *Crr1a^G004^* or *Crr1a^Kinami90_a^* alleles (Fig. 4). These cultivars could be used as genetic resources to breed CR cultivars via marker-assisted selection. Twenty-five and six cultivars, in which the 704-bp and 428-bp were detected by exon 1 markers (Crr1a_exon1_F and Crr1a_exon1_R) and exon 4 markers (Crr1a_exon4_F, Crr1a_exon4_R1, and Crr1a_exon4_R2), respectively, were speculated to possess the susceptible allele. Kawamura *et al.* (2016) predicted clubroot resistance of inbred lines using DNA markers developed based on the sequence differences in exon 4 of *Crr1a* between G004 and A9709, and demonstrated that larger and smaller amplified fragments were associated with susceptibility and resistance, respectively. When this marker was used to detect the *Crr1a* allele isolated in this study, a smaller amplified fragment was obtained from the *Crr1a^Kiko85_a^* allele (data not shown), suggesting the risk of selecting plants with the susceptible *Crr1a* allele. Information on allelic variation would help to develop gene-specific markers to prevent such a risk.

Surprisingly, the two functional *Crr1a* alleles were frequently found in European fodder turnips, but were rarely detected in Japanese CR cultivars (Figs. 4, 5). This result contrasts with that of the other CR genes, *CRa* and/or *CRb* (*CRb^Kato^*), which were probably introduced into many CR cultivars of Chinese cabbage released in Japan (Aruga *et al.* 2013, Kato *et al.* 2013). Because *CRa* and/or *CRb* are known to function as a single dominant gene (Kato *et al.* 2012, Matsumoto *et al.* 1998), the plants heterozygous for this gene can be selected for backcross breeding. In contrast, *Crr1a* is an incomplete dominant gene (Hatakeyama *et al.* 2013). It is possible that the resistance of the heterozygous plants for Crr1a is insufficient, leading to loss of functional alleles during selection. Another possibility is that a certain CR cultivar harboring the functional *Crr1a* allele was used as a genetic resource in CR breeding. However, our findings suggest that *Crr1a* has the potential to increase the level of clubroot resistance in existing CR cultivars. The DNA markers developed in this study could facilitate the efficient and precise selection of plants possessing *Crr1a*.

In conclusion, we demonstrated the sequence variation and functionality of *Crr1a* alleles in *B. rapa*. Gain-of-function analysis using the chimeric genes revealed that the C-terminal region of the *Crr1a* gene is essential for clubroot resistance. We discovered another functional allele, developed DNA markers for the functional alleles, and showed their distribution in CR F_1_ cultivars of Chinese cabbage. These results would contribute to CR breeding via marker-assisted selection and help our understanding of the molecular mechanisms underlying clubroot resistance.

## Author Contribution Statement

KH conceived the idea and designed the study. KH and SY conducted the experiments, analyzed the data, and wrote the manuscript. YT, KT, and SM provided advice on the experimental implementation and helped to draft the manuscript. All authors read and approved the manuscript.

## Supplementary Material

Supplemental Figures

Supplemental Tables
