# Supplementary material for: Allelic variation of a clubroot resistance gene (Crr1a) in Japanese cultivars of Chinese cabbage (Brassica rapa L.)
Source: Breed Sci. 2022 Mar 5;72(2):115–23. doi: 10.1270/jsbbs.21040 (PMC9522534; doi:10.1270/jsbbs.21040)
Supplement: Supplementary file 1 — Supplemental Figures [file 72_115_s1.pdf]

Hatakeyama et al./Breeding Science
doi: 10.1270/jsbbs.21040

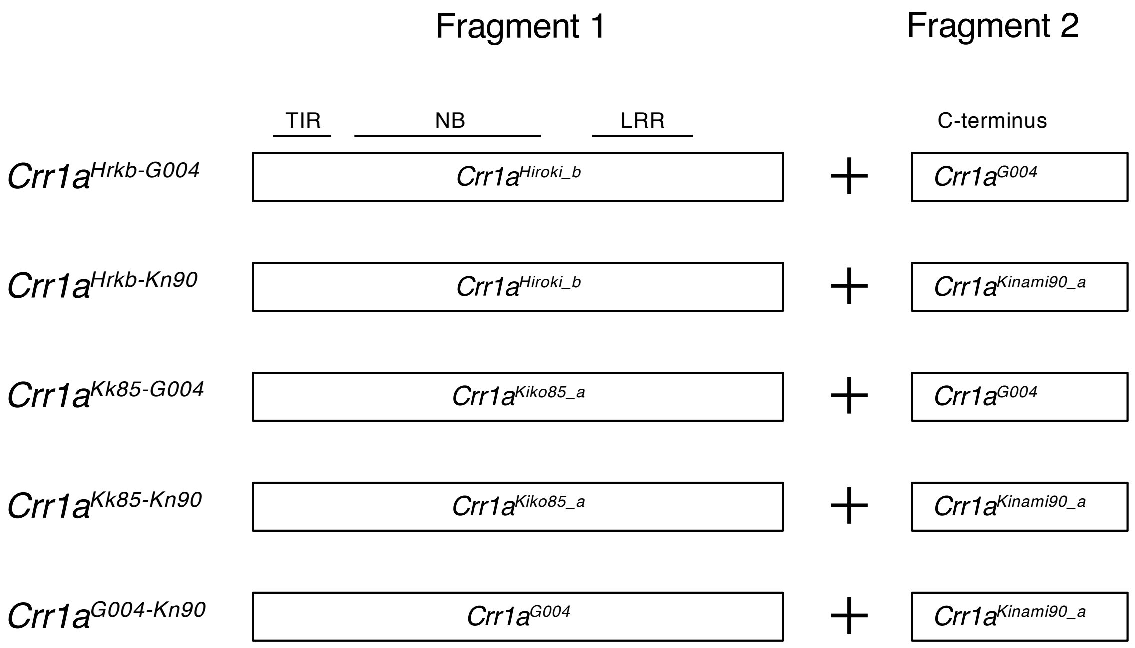

Supplemental Fig. 1. Schematic representation of chimeric *Crr1a* genes used in this study.

```
Crr1a_G004         1 DVFPSFRGEDVRIGFLSHIQKEFKRKGITPFIDNEIRRGESIGPE  45
Crr1a_Hiroki_b     1 DVFPSFRGEDVRIGFLSHIQKEFKRKGITPFIDNEIRRGESIGPE  45
Crr1a_Kiko85_a     1 DVFPSFRGEDVRIGFLSHIQKEFKRKGITPFIDNEIRRGESIGPE  45
Crr1a_Kinami90_a   1 DVFPSFRGEDVRIGFLSHIQKEFKRKGITPFIDNEIRRGESIGPE  45
                     *********************************************

Crr1a_G004        46 LIRAIRGSKIAIVLLSRNYASSKWCLDELVEVMKCKEELGQTVIP  90
Crr1a_Hiroki_b    46 LIRAIRGSKIAIVLLSRNYASSKWCLDELVEVMKCKEELGQTVIP  90
Crr1a_Kiko85_a    46 LIRAIRGSKIAIVLLSRNYASSKWCLDELVEVMKCKEELGQTVIP  90
Crr1a_Kinami90_a  46 LIRAIRGSKIATILLSRNYASSKWCLDELVEIMKCKEELGQTVIP  90
                     ***********  ****************** *************

Crr1a_G004        91 VFYKVDPSHVKKLRGYFGKVFEKTCEGKSKEDTEKWRHAL 130
Crr1a_Hiroki_b    91 VFYKVDPSHVKKLRGYFGKVFEKTCEGKSKEDTEKWRHAL 130
Crr1a_Kiko85_a    91 VFYKVDPSHVKKLRGYFGKVFEKTCEGKSKEDTEKWRHAL 130
Crr1a_Kinami90_a  91 VFYKVDPSDVKKLRGYFGKVFEKTCEGKSKEDTEKWRHAL 130
                     ******** *******************************
```

Supplemental Fig. 2. Alignment of the deduced TIR domain sequences of *Crr1a* alleles. Asterisks below the sequence indicate identical amino acids among 4 alleles.

```
Crr1a_G004          1 DNEAAMIEQIATDVSNKLISSVPSSDFNSLVGMRAHMKSMELLLR  45
Crr1a_Hiroki_b      1 DNEAAMIEQIATDVSNKLISSVPSSDFNSLVGMRAHMKSMELLLR  45
Crr1a_Kiko85_a      1 DNEAAMIEQIATDVSNKLISSVPSSDFNSLVGMRAHMKSMELLLR  45
Crr1a_Kinami90_a    1 DNEAAMIEQIATDVSNKLISSVPSSDFDSLVGMRAHMKSMELLLR  45
                      **************************** ****************
                                   P-loop
Crr1a_G004         46 LDSDEVRMIGIWGPSGIGKSTIARSLFSQHSPDFQLSVFMENIKR  90
Crr1a_Hiroki_b     46 LDSDEVRMIGIWGPSGIGKSTIARSLFSQHSPDFQLSVFMENIKR  90
Crr1a_Kiko85_a     46 LDSDEVRMIGIWGPSGIGKSTIARSLFSQHSPDFQLSVFMENIKR  90
Crr1a_Kinami90_a   46 LDSDEVRMIGIWGPSGIGKSTIARSLFSQHSPDFQLSVFMDNIKR  90
                      *************************************** *****

Crr1a_G004         91 EYPRPCFDRYSAQVQLQNKFLSLILNQNDVAIHHLGVAQDRLKNK 135
Crr1a_Hiroki_b     91 EYPRPCFDRYSAQVQLQNKFLSLILNQNDVAIHHLGVAQDRLKNK 135
Crr1a_Kiko85_a     91 EYPRPCFDRYSAQVQLQNKFLSLILNQNDVAIHHLGVAQDRLKNK 135
Crr1a_Kinami90_a   91 EYPRPCFDRYSAQVQLQNKFLSLILNQNDVAIHHLGVAQDRLKNK 135
                      *********************************************
                        Kinase-2            RNBS-B
Crr1a_G004        136 KVLVVLDDVDHSAQLDALAKETCWFGSGSRIIVTTQDKKILNAHR 180
Crr1a_Hiroki_b    136 KVLVVLDDVDHSAQLDALAKETCWFGSGSRIIVTTQDKKILNAHR 180
Crr1a_Kiko85_a    136 KVLVVLDDVDHSAQLDALAKETCWFGSGSRIIVTTQDKKILNAHR 180
Crr1a_Kinami90_a  136 KVLVVLDDVDHSAQLDALAKETCWFGSGSRIIVTTQDIRILNAHR 180
                      ************************************  *******
                                                          GLPL
Crr1a_G004        181 INHIYEVGFPHDDEALEIFCINAFGQKSPYDGFGDLAREVTRLVG 225
Crr1a_Hiroki_b    181 INHIYEVGFPHDDEALEIFCINAFGQKSPYDGFGDLAREVTRLVG 225
Crr1a_Kiko85_a    181 INHIYEVGFPHDDEALEIFCINAFGQKSPYDGFGDLAREVTRLVG 225
Crr1a_Kinami90_a  181 INHIYKVGFPHDDEALEIFCINAFGQKSPYDGFRNLAREVTRLVG 225
                      ***** ***************************  **********

Crr1a_G004        226 NLPLGLSVMGSYFKGLSKEVWERELPRLRTRLDGETESILKFSYD 270
Crr1a_Hiroki_b    226 NLPLGLSVMGSYFKGLSKEVWERELPRLRTRLDGETESILKFSYD 270
Crr1a_Kiko85_a    226 NLPLGLSVMGSYFKGLSKEVWERELPRLRTRLDGETESILKFSYD 270
Crr1a_Kinami90_a  226 KLPLGLSVMGSYFKGLSKEVWERELPRLRTRLDGETESILKFSYD 270
                       ********************************************

Crr1a_G004        271 ALCDEDQALFLHIACFFNGERTDKVEEFLAEKFVAVEGRLRVLAE 315
Crr1a_Hiroki_b    271 ALCDEDQALFLHIACFFNGERTDKVEEFLAEKFVAVEGRLRVLAE 315
Crr1a_Kiko85_a    271 ALCDEDQALFLHIACFFNGERTDKVEEFLAEKFVAVEGRLRVLAE 315
Crr1a_Kinami90_a  271 ALCDEDQALFLHIACFFKGERIDKVEEFLAEKFVAVEGRLRVLAE 315
                      *************** *** *************************
                                    MHDV
Crr1a_G004        316 KSLISVGSEGYIRMHDLLARLGREIVRKQSPNEPGQ 351
Crr1a_Hiroki_b    316 KSLISVGSEGYIRMHDLLARLGREIVRKQSPNEPGQ 351
Crr1a_Kiko85_a    316 KSLISVGSEGYIRMHDLLARLGREIVRKQSPNEPGQ 351
Crr1a_Kinami90_a  316 KSLISVDSEGYIRMHDLLARLGREIVRKQSPNEPGQ 351
                      ****** *****************************
```

Supplemental Fig. 3. Alignment of the deduced NB domain sequences of *Crr1a* alleles. Conserved motifs characteristic of the NB domains are shown above the sequences.

```
Crr1a_G004         1 NKTIRNLKWMDLSHSKNLKELPN   23
Crr1a_Hiroki_b     1 NKTIRNLKWMDLSHSKNLKELPN   23
Crr1a_Kiko85_a     1 NKTIRNLKWMDLSHSKNLKELPN   23
Crr1a_Kinami90_a   1 NKTIRNLKWMDLSHSKNLKELPN   23
                     ***********************

Crr1a_G004        24 LSTATNLRELNLFGCSSLMELPSS  47
Crr1a_Hiroki_b    24 LSTATNLRELNLFGCSSLMELPSS  47
Crr1a_Kiko85_a    24 LSTATNLRELNLFGCSSLMELPSS  47
Crr1a_Kinami90_a  24 LSTATNLRELGLFGCSSLMELPSS  47
                     ********** *************

Crr1a_G004        48 IGNLTNLKKLNLKLCSSLMELPSS  71
Crr1a_Hiroki_b    48 IGNLTNLKKLNLKLCSSLMELPSS  71
Crr1a_Kiko85_a    48 IGNLTNLKKLNLKLCSSLMELPSS  71
Crr1a_Kinami90_a  48 IGNLTNLKKLNLKLCSSLMELPSS  71
                     ************************

Crr1a_G004        72 IGNMTNLENLNLSGCSSLVELPSS  95
Crr1a_Hiroki_b    72 IGNMTNLENLNLSGCSSLVELPSS  95
Crr1a_Kiko85_a    72 IGNMTNLENLNFSGCSSLVELPSS  95
Crr1a_Kinami90_a  72 IGNMTNLENLNLSGCSSLVELPSS  95
                     *********** ************

Crr1a_G004        96 ISNMTNLENFNLSQCSSVVRLSFS 119
Crr1a_Hiroki_b    96 ISNMTNLENFNLSQCSSVVRLSFS 119
Crr1a_Kiko85_a    96 ISNMTNLENFNLSQCSSVVRLSFS 119
Crr1a_Kinam90_a   96 ISNMTNLEKLNLSQCSSVVRLSFS 119
                     ********  **************

Crr1a_G004       120 IGNMTNLKELELNECSSLVELT   141
Crr1a_Hiroki_b   120 IGNMTNLKELELNECSSLVELT   141
Crr1a_Kiko85_a   120 IGNMTNLKELELNECSSLVELT   141
Crr1a_Kinami90_a 120 IGNMTNLKELDLSECSSLVELT   141
                     ********** * *********

Crr1a_G004       142 FGNMTNLKNLDPNRCSSLVEISSS 165
Crr1a_Hiroki_b   142 FGNMTNLKNLDPNRCSSLVEISSS 165
Crr1a_Kiko85_a   142 FGNMTNLKNLDPNRCSSLVEISSS 165
Crr1a_Kinam90_a  142 FGNMTNLKNLDLSGCSSLVEISSS 165
                     ***********   **********

Crr1a_G004       166 IGNMTNLVRLDLTGCSSLVELPYS 189
Crr1a_Hiroki_b   166 IGNMTNLVRLDLT----------- 178
Crr1a_Kiko85_a   166 IGNMTNLVRLDLTGCSSLVELPYS 189
Crr1a_Kinami90_a 166 IGNMTNLVKLDLS----------- 178
                     ******** ***

Crr1a_G004       190 IGNMTNLETLELSGCSSLVELPSS 213
Crr1a_Hiroki_b   179 -------------GCSSLVELPSS 189
Crr1a_Kiko85_a   190 IGNMTNLETLELSGCSSLVELPSS 213
Crr1a_Kinami90_a 179 -------------RCSSLEELPSS 189
                                   **** *****

Crr1a_G004       214 IGNLHNLKRLNLRNCSTLMALPV  236
Crr1a_Hiroki_b   190 IGNLHNLKRLNLRNCSTLMALPV  212
Crr1a_Kiko85_a   214 IGNLHNLKRLNLRNCSTLMALPV  236
Crr1a_Kinami90_a 190 IGNMTNLENLNLGNCSKLMSLPV  212
                     ***  **  *** *** ** ***

Crr1a_G004       237 NINMKSLDFLDLSYCSVLKSFPEI 260
Crr1a_Hiroki_b   213 NINMKSLDFLDLSYCSVLKSFPEI 236
Crr1a_Kiko85_a   237 NINMKSLDFLDLSYCSVLKSFPEI 260
Crr1a_Kinami90_a 213 NINMKYLDILDLSDCSSLKSFPEI 236
                     ***** ** **** ** *******
                     I..M..L..L.L..C..L..LP..
```

Supplemental Fig. 4. Alignment of amino acid sequences of the deduced LRR domains of *Crr1a* alleles. The consensus sequence of LRRs are given at the bottom. The amino acid altered between Crr1a^{G004} and Crr1a^{Kiko85_a} is boxed.

```
Crr1a_G004         1 STNIIFLGIKGTAIEEIPTSIRSWSRLDTLDMSYSENLRKSHHAFDLITN   50
Crr1a_Kinam90_a    1 STNIRVLKLNGTAIEEIPTSIRSWSRLEWLHMSYSENLGKSHHAFDLITN   50
                     ****  *   *****************  * ******* ***********

Crr1a_G004        51 LHLSDTGIQEISPWVKEMSRLRELVINGCTKLVSLPQLPDSLEFMHVENC  100
Crr1a_Kinam90_a   51 LHLSDTGIQEISPLVKEMSRLQTLVINGCTKLVSLPQLPDSLEYINAENC  100
                     ************* *******  ********************    ***

Crr1a_G004       101 ESLERLDSLDCSFYRTKLTDLRFVNCLKLNREAVDLILKTSTKIWAIFPG  150
Crr1a_Kinam90_a  101 ESLERLD---CSFYRTKLTDLRFVNCLKLNREAVDLILKTSTKIWAIFPG  147
                     *******   ****************************************

Crr1a_G004       151 ESVPAYFSYRATGSSVSMKLNRFDTRFPTSLRFKACILLVTNPDDVEPAA  200
Crr1a_Kinam90_a  148 ETVPAYFSYRATGSSVSMKLNGFDTRFPTSLRFKACILLVTNPDDVEPAA  197
                     * ******************* ****************************

Crr1a_G004       201 WYRSDMSYCINGKLRDAGVFLAYTHIWDPLRPRSEHLVVIEFEETVTSPE  250
Crr1a_Kinam90_a  198 WYRSDISYCINGKLRDAGVLLLYSHIWDPLCPRSEHLVVIEFEETVTSPE  247
                     ***** ************* * * ****** *******************

Crr1a_G004       251 LVFEFRFEKENWEIKECGLRPLESLALSC                        279
Crr1a_Kinam90_a  248 LVFEFRFEKKNGEIKECGLRPLESLALSC                        276
                     ********* * *****************
```

Supplemental Fig. 5. Alignment of amino acid sequences of the deduced post-LRR domains of *Crr1a* alleles. Bent arrow indicates the C-terminal region lacking in the susceptible alleles, *Crr1a*$^{Hiroki\_b}$ and *Crr1a*^{Kiko85}.

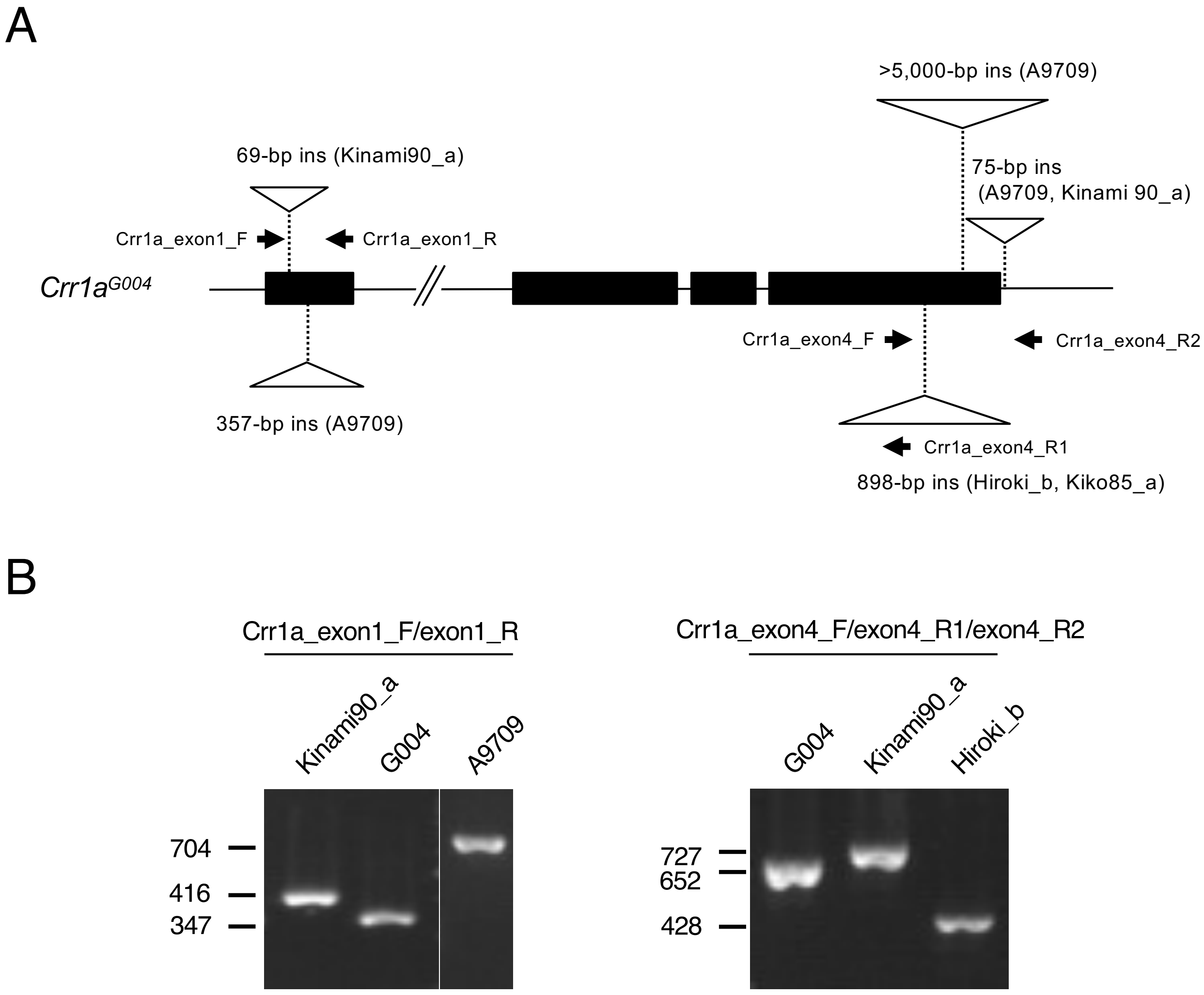

Supplemental Fig. 6. Development of the functional allele specific DNA markers for *Crr1a*
A, Schematic representation of structural differences among *Crr1a* alleles. Size and name of alleles of the insertions found in *Crr1a* alleles other than *Crr1a*^{G004} are shown. Arrows indicate primers used for the detection of the functional alleles. B, Difference of the fragment size obtained from the amplification with primers shown in A. For the detection for *Crr1a*$^{Kinami90\_a}$ and *Crr1a*$^{Hiroki\_b}$, genomic clone was used as a template.
