# Supplementary material for: Allelic variation of a clubroot resistance gene (Crr1a) in Japanese cultivars of Chinese cabbage (Brassica rapa L.)
Source: Breed Sci. 2022 Mar 5;72(2):115–23. doi: 10.1270/jsbbs.21040 (PMC9522534; doi:10.1270/jsbbs.21040)
Supplement: Supplementary file 2 — Supplemental Tables [file 72_115_s2.pdf]

doi: 10.1270/jsbbs.21040

Supplemental Table 1. List of the CR and non-CR cultivars of Chinese cabbage used in this study

CR/non-CR	No. [a)]	Name of cultivar	Seed company
CR	1	Kinami 90	Ishii Seed Growers Co., Ltd.
	2	CR Seiga 65	Ishii Seed Growers Co., Ltd.
	3	SCR Hiroki	Sumika Agrotech Co., Ltd.
	4	SCR Kimi 85	Sumika Agrotech Co., Ltd.
	5	Kougetsu 77	Kaneko Seeds Co., Ltd.
	6	Kisho	Kaneko Seeds Co., Ltd.
	7	Kiryoyoshi 70	Kaneko Seeds Co., Ltd.
	8	Moegi	Kaneko Seeds Co., Ltd.
	9	Shoki	Kaneko Seeds Co., Ltd.
	10	CR Kisen	Tokita Seed Co., Ltd.
	11	CR Saitaikai	Tokita Seed Co., Ltd.
	12	CR Kaio	Tokita Seed Co., Ltd.
	13	CR Kisaku 80	Marutane Co., Ltd.
	14	CR Kyotakara 70	Marutane Co., Ltd.
	15	CR Kyotakara 80	Marutane Co., Ltd.
	16	CR Shinki	Taki & Co., Ltd.
	17	Kukai 65	Taki & Co., Ltd.
	18	Yuki	Taki & Co., Ltd.
	19	Kigokoro 65	Taki & Co., Ltd.
	20	Kigokoro 85	Taki & Co., Ltd.
	21	Kiraboshi	Taki & Co., Ltd.
	22	Kitaboshi 90	Taki & Co., Ltd.
	23	Haregi 65	Taki & Co., Ltd.
	24	Haregi 75	Taki & Co., Ltd.
	25	Kiraku 70	Tohoku Seed Co., Ltd.
	26	Shinshu Daihuku	Tohoku Seed Co., Ltd.
	27	Hukutakara 60	Tohoku Seed Co., Ltd.
	28	Daihuku 206	Tohoku Seed Co., Ltd.
	29	CR Satokaze	Sakata Seed Co.
	30	Minebuki 505	Sakata Seed Co.
	31	Satobuki 613	Sakata Seed Co.
	32	Chiyobuki 70	Sakata Seed Co.
	33	Chiyobuki 85	Sakata Seed Co.
	34	CR Kanko	Nippon Norin Seed Co.
	35	Akiriso	Nippon Norin Seed Co.
	36	Kihuku 65	Nippon Norin Seed Co.
	37	Kien 80	Nippon Norin Seed Co.
	38	SCR Shinriso	Nippon Norin Seed Co.
	39	Akimeki	Nippon Norin Seed Co.
	40	SCR Akinishiki	Nippon Norin Seed Co.
	41	Ryutoku	Watanebe Seed Co., Ltd.
	42	Kiko 85	Watanebe Seed Co., Ltd.
	43	Kunki 65	Watanebe Seed Co., Ltd.
	44	Kunki 80	Watanebe Seed Co., Ltd.
	45	Kiai 65	Watanebe Seed Co., Ltd.
	46	Meikyo	Watanebe Seed Co., Ltd.
	47	W-1117	Watanebe Seed Co., Ltd.
	48	W-1116	Watanebe Seed Co., Ltd.
non-CR	49	Aichi	Taki & Co., Ltd.
	50	Kyoto 3	Taki & Co., Ltd.
	51	Kashin Hakusai	Taki & Co., Ltd.
	52	Shin Azuma	Taki & Co., Ltd.
	53	Muso	Taki & Co., Ltd.
	54	Chihiri 70	Taki & Co., Ltd.
	55	Banki	Taki & Co., Ltd.
	56	Haruwarai	Taki & Co., Ltd.
	57	Cream 2	Watanebe Seed Co., Ltd.
	58	Shinriso	Nippon Norin Seed Co.
	59	Banchu Daihuku	Tohoku Seed Co., Ltd.
	60	Toshikoshi	Sakata Seed Co.

a, Lane No. in Fig. 5

Supplemental Table 2. Sequences of primers used in this study

Primer name	Sequence (5' - 3')	Use
Crr1a_Hirok_InF_F1_F	AGCTTTTTGAAACCCATGAAATTTCAATCGTTTTTGAA	Amplification of the Fragment 1 from G004, SCR Hiroki, and Kiko85
Crr1a_Hirok_InF_F1_R	AGAGCAATCTAGACTATCTAGTCTCTCGAGGGAC	Amplification of the Fragment 1 from G004, SCR Hiroki, and Kiko85
Crr1a_G004_InF_F2_F	AGTCTAGATTGCTCTTTTTAC	Amplification of the Fragment 2 from G004
Crr1a_G004_InF_F2_R	CACCAAACCAGTCGACAGCTTCCATTAACATGAGA	Amplification of the Fragment 2 from G004
Crr1a_Kn90_InF_F2_F	TAGATTGCTCTTTTTACAGGA	Amplification of the Fragment 2 from Kinami90
Crr1a_Kn90_InF_F2_R	CACCAAACCAGTCGACAGTTTCCATCAACATGAGA	Amplification of the Fragment 2 from Kinami90
Crr1a_exon1_F	ATGAAATTTCAATCGTTTTTGAAAG	Detection of the Crr1aKinami_a
Crr1a_exon1_R	AGTTCTGGACCGATGGATTCT	Detection of the Crr1aKinami_a
Crr1a_exon4_F	TGCACAAAGCTGGTTTCGCTC	Detection of the Crr1aG004
Crr1a_exon4_R1	GTTCTGTTTCCGCAAGTCAGACGAA	Detection of the Crr1aG004
Crr1a_exon4_R2	GCATTACTATATTTTATGCACATGCG	Detection of the Crr1aG004
